# Exploiting tRNAs to Boost Virulence

**DOI:** 10.3390/life6010004

**Published:** 2016-01-19

**Authors:** Suki Albers, Andreas Czech

**Affiliations:** Biochemistry and Molecular Biology, Department of Chemistry, University of Hamburg, Martin-Luther-King-Platz 6, Hamburg 20146, Germany; suki.albers@chemie.uni-hamburg.de

**Keywords:** virus, tRNA, tRNA-like elements (TLE), codon usage, modification

## Abstract

Transfer RNAs (tRNAs) are powerful small RNA entities that are used to translate nucleotide language of genes into the amino acid language of proteins. Their near-uniform length and tertiary structure as well as their high nucleotide similarity and post-transcriptional modifications have made it difficult to characterize individual species quantitatively. However, due to the central role of the tRNA pool in protein biosynthesis as well as newly emerging roles played by tRNAs, their quantitative assessment yields important information, particularly relevant for virus research. Viruses which depend on the host protein expression machinery have evolved various strategies to optimize tRNA usage—either by adapting to the host codon usage or encoding their own tRNAs. Additionally, several viruses bear tRNA-like elements (TLE) in the 5′- and 3′-UTR of their mRNAs. There are different hypotheses concerning the manner in which such structures boost viral protein expression. Furthermore, retroviruses use special tRNAs for packaging and initiating reverse transcription of their genetic material. Since there is a strong specificity of different viruses towards certain tRNAs, different strategies for recruitment are employed. Interestingly, modifications on tRNAs strongly impact their functionality in viruses. Here, we review those intersection points between virus and tRNA research and describe methods for assessing the tRNA pool in terms of concentration, aminoacylation and modification.

## 1. Introduction

Protein biosynthesis is effected by ribosomes that translate the information in a mRNA by scanning along it. By recruiting ternary complexes containing eEF1A, GTP and a specific aminoacylated transfer RNA (tRNA) bearing an anticodon that is complementary to the codon on the mRNA, ribosomes incorporate the cognate amino acid into the growing polypeptide chain. So far, no mechanism for the active transport of ternary complexes to the ribosomes is known. Rather, ribosomes and ternary complexes diffuse through the crowded molecular environment of cells and meet stochastically. Since eEF1A is highly abundant, formation of the ternary complex is not rate limiting. Thus, the rate of translation of a particular codon correlates with the tRNA concentration [1], *i.e.*, codons decoded by highly abundant tRNAs are generally translated faster than codons decoded by tRNAs of low abundance. It has been shown that the speed of gene expression can be optimized by selecting codons for highly abundant tRNAs [2]. However, the slow translation of some mRNA regions is crucial for proper co-translational folding of corresponding protein regions, as well as protein trafficking, and consequently for correct function [3]. The measurement of tRNA concentrations is crucial in relation to predicting the speed of translation (reviewed in [4]). Such measurements are possible on the global scale with tRNA microarrays, which can also allow the aminoacylation status of tRNAs to be assessed [5,6,7]. Most recently, deep sequencing has also proved helpful [8,9].

## 2. Codon Usage and tRNA Concentrations

Viruses depend on the host translation machinery for the synthesis of their own proteins [10]. Therefore, it is expected that there is a selective pressure for the virus to adjust the codon usage to that of the corresponding host organism in order to enable the efficient translation of viral proteins [11]. This has been shown to be especially the case for highly-expressed genes, *i.e.*, those coding for proteins involved in DNA packaging or structural proteins [12,13]. Indeed, several studies have shown that optimization of viral codon usage towards the codon repertoire of the host results in an enhancement of translation [14,15,16,17]. e.g., a pattern of codon usage similar to that of their host has been observed for the positive-strand poliovirus (PV) and foot-and-mouth disease virus (FMDV), both members of the picornaviridae [18,19]. Similarity of the codon usage between virus and host, however, results in competition for tRNAs for protein synthesis. Viruses have evolved different ways to evade this competition. For example, PV and FMDV inhibit cap-dependent translation of host mRNA by viral-encoded proteinase 2A (PV) and proteinase L (FMDV). These proteinases cleave the eukaryotic initiation factor 4G (eIF4G), a component of the cap-binding complex eIF4F, leading to the limitation of cap-dependent host protein synthesis. However, the cleavage product of eIF4G is still able to function in the internal ribosome entry site (IRES)-mediated initiation of translation of viral proteins in PV and FMDV [20,21,22,23]. Another way to prevent competition for tRNAs and also to broaden the spectrum of host cells is to evolve a pattern of codon usage that differs from that of the host. Several viruses encode their own tRNAs as a way of compensating for low levels of host tRNAs corresponding to codons that the viruses use with high frequency in their own mRNAs. The phenomenon of virally encoded tRNAs was first discovered four decades ago in a group of DNA bacteriophages [24,25]. The first viruses identified to encode their own set of tRNAs have been the bacteriophage T4 which possesses eight tRNA genes and the bacteriophage T5 carrying at least 14 different tRNAs [24,26,27,28,29,30]. The occurrence of tRNA genes is not restricted to bacteriophages; tRNAs are widespread in different families of viruses. However, double stranded (ds) DNA viruses are so far the only identified class of viruses encoding tRNAs [31]. dsDNA viruses, especially large ones such as the mimiviridae or phycodnaviridae, contain a sizeable repertoire of tRNAs [32]. The marine cyanophages myoviridae and siphoviridae encode up to 41 tRNAs per genome. The cyanophage-encoded tRNAs include the initiator tRNA^fMet^ and a range of species bearing anticodons that match all 20 amino acid specificities [33,34]. The most studied viral-encoded tRNAs belong to phycodnaviruses infecting chlorella green algae. Some chlorella viruses (phycodnaviridae) encode up to 16 tRNAs [35] and in fact many of these tRNAs in the infected cells have been found to be aminoacylated at their 3′-termini, suggesting their usage in viral protein synthesis [36]. A summary of the tRNA species encoded by different viruses is shown in Table 1.

The murine gammaherpesvirus 68 (MHV-68)- and chlorella virus-encoded tRNA genes contain internal A and B box sequences that serve as promoter elements for eukaryotic RNA polymerase III [36,37,38]. In eukaryotes, both the A and B boxes are recognized by the transcription factor IIIC (TFIIIC) and direct the binding of TFIIIB ~50 bp upstream of the transcription start site. Besides the internal promoters the upstream region is presumed to play a role in regulation of tRNA transcription [39]. Consequently, TFIIIB recruits RNA polymerase III to the DNA and transcription is initiated [40]. How are virus-encoded tRNAs synthesized? The paramecium bursaria chlorella virus 1 (PBCV-1) genome does not encode an RNA polymerase but encodes the transcription factors TFIIB, TFIID and TFIIS [41,42]. This suggests that the host RNA polymerase is responsible for the transcription of viral tRNA genes in conjunction with viral transcription factors [36]. However, there is so far no evidence, that the viral tRNA-encoding genes may be transcribed by the host RNA polymerase III. In the case of mimivirus, tRNAs with polyadenylated 3′-termini have been observed. Therefore, the assumption arises that mimivirus tRNA genes are transcribed by the viral-encoded RNA polymerase II transcription machinery [43]. The tRNA genes of chlorella virus kyoto 2 (CVK2) are transcribed polycistronically as large precursor RNAs and subsequently processed to generate mature tRNA [36]. It has been suggested that ribonuclease III (RNase III) is involved in the processing of phydonaviridae precursor tRNA molecules [38,44]. RNase III enzymes have been found in both prokaryotic and eukaryotic cells and their main role is the processing of large precursor molecules to generate ribosomal RNA (rRNA) as well as tRNAs. In prokaryotes, such as *E. coli*, several operons for rRNAs are distributed throughout the genome. Each polycistronic operon contains 23S, 16S and 5S rRNA coding sequences interspersed with a variable number of tRNA genes. These operons are transcribed as large multimeric precursor molecules which are consequently cleaved at double-stranded stem regions by RNase III to generate the individual rRNAs as well as tRNAs which then mature via further processing at their 3′- and 5′-termini [45,46]. It has been shown, that *E. coli* RNase III is involved in the maturation of bacteriophage T4-encoded tRNAs [47,48]. A similar function has been suggested for an RNase III homolog expressed at the early stage of infection by the chlorella viruses ATCV-1, CVK2 and PBCV-1 [36,38,44]. The a464r open reading frame of PBCV-1 is conserved in chlorella infecting viruses with an amino acid sequence that is about 30%–35% identical to RNase III from *E. coli* [38].

**Table 1:** Number and identity of the transfer RNA (tRNA) species encoded by different viruses.

Virus Family	Virus	Genome Size [bp]	tRNA Species	No. of tRNAs	Reference
Myoviridae	Bacterio-phage T4	168,903	tRNA^Ile^_GAU_, tRNA^Ile^_CAU_, tRNA^Arg^_UCU_, tRNA^Leu^_UAA_, tRNA^Ser^_UGA_, tRNA^Gly^_UCC_, tRNA^Pro^_UGG_, tRNA^Thr^_UGU_, tRNA^Gln^_UUG_	8	[29]
Siphoviridae	Bacterio-phage T5	121,752	tRNA^Ile1^, tRNA^Ile2^, tRNA^Arg^, tRNA^Leu^, tRNA^Ser1^, tRNA^Ser2^, tRNA^Gly^, tRNA^Pro^, tRNA^Thr^, tRNA^Gln^, tRNA^His1^, tRNA^His2^, tRNA^Met^, tRNA^fMet^, tRNA^Lys^, tRNA^Val^, tRNA^Ala^, tRNA^Phe^, tRNA^Asn^, tRNA^Cys^, tRNA^Glu^, tRNA^Trp^, tRNA^Tyr^	>14	[26,27,30,49,50]
Mimiviridae	Acantha-moeba polyphagamimivirus	1,181,404	tRNA^Leu^_UAA_, tRNA^Leu^_UAA2_, tRNA^Leu^_CAA_, tRNA^Trp^_CCA_, tRNA^His^_GUG_, tRNA^Cys^_ACA_	6	[51,52]
Herpesviridae	MHV 68 ^a^	118,237	Identified: tRNA^Val^_AAC_, tRNA^Met^_CAU_, tRNA^Thr^_AGU_	8 ^b^	[53]
Phycodna-viridae	PBCV-1 ^a^	330,740	tRNA^Lys^_CUU_, tRNA^Lys^_CUU2_, tRNA^Lys^_UUU_, tRNA^Asn^_GUU_, tRNA^Asn^_GUU2_, tRNA^Leu^_CAA_, tRNA^Leu^_UAA_, tRNA^Arg^_UCU_, tRNA^Ile^_UAU_, tRNA^Tyr^_GUA_, tRNA^Val^_AAC_	11	[36]
	ATCV-1 ^a^	288,047	tRNA^Asn^_GUU_, tRNA^Asn^_GUU2_, tRNA^Val^_AAC_, tRNA^Val^_AAC2_, tRNA^Lys^_CUU_, tRNA^Arg^_UCU_, tRNA^Leu^_UAA_, tRNA^Asp^_GUC_, tRNA^Gly^_UCC_, tRNA^Tyr^_GUA_, tRNA^Ser^_ACU_	11	[44]
	CVK 2 ^a^	330,000–380,000	tRNA^Lys^_CUU_, tRNA^Lys^_CUU2_, tRNA^Lys^_UUU_, tRNA^Asn^_GUU_, tRNA^Asn^_GUU2_, tRNA^Leu^_UAA_, tRNA^Leu^_CAA_, tRNA^Arg^_UCU_, tRNA^Asp^_GUC_, tRNA^Gly^_UCC_, tRNA^Gln^_CUG_, tRNA^Ile^_UAU_, tRNA^Tyr^_GUA_, tRNA^Val^_AAC_	14	[36]

^a^ MHV 68 corresponds to the murine gammaherpesvirus 68, PBCV-1 to paramecium bursaria chlorella virus 1, ATCV-1 to acanthocystis turfacea chlorella virus 1 and CVK2 to chlorella virus Kyoto 2; ^b^ From the eight putative tRNA genes in the MHV 68 genome, only three possess a 7 nt anticodon loop and could be attributed to a predicted anticodon and amino acid sequence specificity.

Chlorella viruses were the first viruses known to encode a component of the translation machinery other than tRNAs. Their genes encode a homolog of the eukaryotic translation elongation factor 3 (eEF3), which is crucial for translation elongation in yeast and higher fungi [54,55]. The fungal eEF3, which is absent in other eukaryotic organisms, facilitates the release of deacylated tRNAs from the ribosomal E-site and thus allows binding of ternary complexes to the A site [56]. The amino acid sequence of the PBCV-1-encoded homolog is 45% identical to yeast eEF3 and may serve a role in facilitating the translation of viral mRNAs [54]. Mimiviruses have been shown to encode nine translation factors analogous to eukaryotic ones (initiation factors eEIF1, eEF4A, eIF4E; elongation factor eEF1A and termination factor eRF1) as well as four aminoacyl-tRNA synthetases [57]. In the case of mimivirus, it has been suggested that these translation-related genes are relics of a more complex ancestral protein translation apparatus from which genes were progressively lost under selection pressure for a reduction of genome size [57]. In the case of dsDNA phages, tRNA genes have been thought to be the result of recruitment from host chromosomes or from recombination with other phages co-infecting bacterial cells. Adaptive selection is then expected to have mediated the retention of tRNAs corresponding to codons that are rare in the host but abundant in phage genes [31]. *In vitro* studies using T4 phage mutants defective in viral-encoded tRNA genes have demonstrated lower burst sizes and rates of protein synthesis. However, the T4 phages were still able to replicate [58]. Thus, the supplementation of T4-encoded tRNAs to the host tRNA pool seems to increase the fitness of phages. In strains of bacteria other than *E. coli* B, the deletion of T4 phage tRNAs diminishes the viral replication [59]. Depletion of tRNAs, which are necessary for viral protein expression is also a host defense mechanism, albeit fatal. Upon T4 bacteriophage infection, host cells express anticodon nuclease (ACNase), which specifically cleaves tRNA^Lys^ and thus inhibits late bacteriophage T4 protein expression [60]. T4 bacteriophage evolved two enzymes to repair cleaved tRNAs to save the life of its host and itself—polynucleotide kinase (PNK) and RNA ligase [61]. Recently, it was found that upon respiratory syncytial virus infection the endonuclease angiogenin is activated leading to specific tRNA cleavage [62,63].

## 3. tRNA-Like Structures (TLS)

The genomes of positive-strand plant viruses function directly as mRNAs in infected cells. Many of these viral genomes, including those of tobamoviruses, tobraviruses, tymoviruses, furo-like virus, hordeiviruses and cucumoviruses, have been shown to possess tRNA-like structures (TLS) in the 3′-untranslated region [32,64,65,66,67,68,69]. Features of the plant viral 3′-tRNA-like structures resembling canonical tRNAs include the aminoacylation of these structural elements via aminoacyl-tRNA synthetases provided by the translation system of the host. Three different amino acid specificities have been shown for the 3′-TLSs: aminoacylation with valine (tymoviruses, furo-like virus, sunnhemp mosaic virus) [70,71], histidine (tobamoviruses) [72,73] as well as tyrosine (bromoviruses, cucumoviruses, hordeiviruses) [32,74,75]. For turnip yellow mosaic virus (TYMV) the kinetics of the 3′-TLS valylation by valyl-tRNA synthetase are similar to that of the canonical tRNA^Val^ in wheat germ extracts and depend on the same anticodon loop identity elements [65,70,71,76]. Intriguingly, aminoacylation of the TYMV 3′-TLS has been shown to be crucial for viral infectivity [77]. Some 3′-TLSs have been shown to possess the tRNA-like L-shaped tertiary structure [78,79,80]. However, the formation of the L-shaped structure is distinct from canonical tRNAs in that different intramolecular interactions exist which enable the 3′-TLS to switch between two different conformations [78]. Some of the 3′-tRNA-like structures form a ternary complex with eEF1A and GTP, required for the elongation reaction of translation in eukaryotes, and are able to interact with ribosomes [81,82,83,84,85]. 

The discovery of 3′-TLSs started in the 1970s with the identification of these structural elements in the genome of TYMV, a member of the tymoviridae family [71,86]. Since then, various putative roles have been claimed for the plant viral 3′-TLS, including a function analog to that of the poly(A) tail. The efficient initiation of translation of eukaryotic mRNAs in the cap-dependent pathway requires the formation of a closed loop structure, in which translation initiation factors mediate the required proximity of the 3′- and 5′-termini of the mRNA. The closed loop structure is achieved via eIF4G-mediated interaction of the poly(A) binding protein (PABP) associated with the 3′-poly(A) tail of the mRNA and the translation initiation factor 4E (eIF4E), a component of the eIF4F complex bound to the 5′-cap. Upon formation of the closed loop structure, the interaction of the small ribosomal subunit with eIF3 bound to eIF4G leads to its association with the 5′-cap and consequent scanning of the mRNA in search of the AUG start codon. In addition to promoting translation initiation, the poly(A) tail functions in stabilizing the RNA by preventing nucleases from degrading it [87,88]. About 80% of plant positive strand viral RNA genomes lack either the 5′-cap, the poly(A) tail or, less commonly, both [89]. In these cases, the poly(A) tail is replaced by viral 3′-tRNA-like structures [90,91,92]. For TYMV, the circularization of the genomic RNA [93] as well as an enhancement of viral translation is mediated by the 3′-TLS together with the 5′-cap [90,91,94]. It has also been suggested that aminoacylated tobacco mosaic virus (TMV) [84] and TYMV 3′-TLS forms a ternary complex with eEF1A and GTP [65,94]. In contrast, the 3′-aminoacylation of the brome mosaic virus (BMV) TLS causes the dissociation of GTP from the binary complex with eEF1A and thus is not able to form a stable ternary complex [81,95]. The interaction of the TYMV 3′-TLSs with eEF1A might lead to a communication with the eIF4F initiation factors bound to the 5′-cap, analogous to the PABP-eIF4G interaction in the case of polyadenylated mRNA, and thereby produce the closed loop structure of the viral genomic RNA [96]. Thus, TYMV 3′-TLS is expected to serve as a translational enhancer as well as a stabilizer of the mRNA. A requirement for the 3′-TLS-mediated translational enhancement is the presence of the aminoacylated 3′-CCA end [32,94]. However, the identity of the amino acid attached to the TYMV 3′-TLS, which is governed by the TLS-anticodon and can thus be mutated, does not affect the ternary complex formation [94].

In addition, 5′, *i.e.*, upstream of viral open reading frames tRNA-like structures can be found as parts of internal ribosome entry sites (IRES). Namely, IRES from the taura syndrome virus and the cricket paralysis virus mimic tRNA structures to interact directly with the ribosome [97,98]. Consequently, translation can be initiated independently from any canonical initiation factors including eIF2 and initiator tRNA^Met^ [99]. Besides this most independent group 1 IRES structures, group 2–4 IRES need varying numbers of translation initiation factors and the question arises whether those also employ tRNA mimicry (reviewed in [100]). To search for tRNA-like structures, Gómez and coworkers used an *in vitro* cleavage assay with RNase P, the ribonuclease responsible for pre-tRNA leader removal, which has been shown to recognize structural elements rather than a particular sequence [101,102,103]. RNase P cleavage was found downstream of the hepatitis C virus IRES [102,103] and an L-shaped tRNA-like structure was confirmed by cryo-electron microscopy [104]. However, since RNase P usually cleaves upstream of tRNA structure in the tRNA precursor and since the enzyme has a broad substrate spectrum including RNAs with no structural similarity to tRNAs, RNase P cleavage experiments should be interpreted cautiously [100].

## 4. tRNAs in Packaging and Priming

For retroviruses, plant pararetroviruses and retrotransposons particular tRNAs serve the very specific function of priming reverse transcription of viral RNA [105]. Reverse transcription generates complementary DNA (cDNA) that can be integrated into the host genome as double stranded DNA. With its own genome integrated in the host’s genetic material, the virus’s entire genetic specification is passed on to daughter cells upon cell division, where it can be activated, multiplied and distributed in new virus particles.

Different viruses use different host tRNAs to prime reverse transcription. The systematics of human endogenous retroviruses (HERVs), which are associated with different cancers, multiple sclerosis and schizophrenia, are based on the tRNA specificity. Different HERVs use 20 different tRNAs to prime reverse transcription, including tRNA^Val^ and tRNA^Trp^ in HERV-V and HERV-W, respectively (reviewed in [106]). Among exogenous retroviruses, the human immunodeficiency virus (HIV) uses tRNA^Lys3^, the rous sarcoma virus (RSV) uses tRNA^Trp^ and murine moloney leukemia virus (MuLV) uses tRNA^Pro^ (reviewed in [105]). The primary interaction appears to be between the 3′-terminus of the tRNA with the primer binding site (PBS) in the 5′-UTR of the viral RNA. In HIV-1, there are additional interactions at two other sites: the anticodon loop interacts with the poly(A) loop and the variable loop interacts with the C-rich region, both in the upper PBS stem of the viral RNA [107]. Of course, RNA:RNA interactions require single-stranded partners to form intermolecular base pairs. However, the tRNA as well as the 5′-UTR of the viral RNA are involved in intramolecular double-stranded stem structures. Thus, those intramolecular base pairs have to be melted to allow intermolecular interactions. The unprocessed Gag-protein as well as the mature nucleocapsid product have RNA chaperone activity and are crucial for annealing of tRNA^Lys^ to the viral RNA [108,109]. However, as has been shown by *in virio* SHAPE analysis of protease-deficient, (*i.e.*, nucleocapsid (Ncp7)-deficient) HIV-1, Gag can only catalyze PBS:anti-PBS interactions. Ncp7 is crucial for annealing tRNA^Lys^ to the regions upstream of the PBS [107]. For MuLV, a chaperoning mechanism for nucleocapsid has been proposed, based on NMR measurements [110]. In contrast to other RNA chaperones, instead of global destabilizing to access a higher energy conformation, nucleocapsid causes local destabilization at strategic points. These points are very specific and are located outside annealing areas. Thus, nucleocapsid binding does not interfere with binding and action of the reverse transcriptase enzyme [111]. Annealing of tRNA is not only crucial for priming reverse transcription, but initiates dimerization of two viral RNA molecules, a prerequisite for packaging them into budding viral particles (virions) [112,113]. In this case, chaperoning activity of Gag is sufficient, allowing PBS:anti-PBS interactions [110]. Those lead to a shift from the long distance interaction (LDI), in which the dimerization site (DIS) is buried in base-pairing interactions, to the branched multiple-hairpin (BMH) conformation [114]. In the latter conformation DIS loops are single-stranded and thus free to interact with each other to form first basepairs between two viral RNA molecules. This kissing-loop interaction is followed by extension of the dsRNA to form a more stable dimer, which is packed in virions [112,113].

Since, as described above, the primer tRNA is very crucial in the life cycle of retroviruses, many of them are actively recruited into newly assembling viral particles. Thus, it has been found that there is a 10-fold enrichment of primer tRNA^Lys^ in HIV-1 virions and a 22-fold enrichment of tRNA^Trp^ in RSV virions over cytosolic concentrations [115]. The most obvious molecule to use in the recruitment of specific tRNA isoacceptors is the respective aminoacyl-tRNA synthetase (aaRS). Hence, HIV-1 and RSV selectively pack lysyl-tRNA synthetase (LysRS) and tryptophanyl-tRNA synthetase (TrpRS) with the cognate tRNA, respectively, into their virions [115]. The conclusion, that this is the way to recruit primer tRNAs in the virus particle is underscored by two findings. First, the number of tRNA and aminoacyl-tRNA synthetase molecules per virus particle is very similar, e.g., 20–25 tRNA^Lys^ molecules [116] and 25 ± 9 lysyl-tRNA synthetase molecules [115]. Second, overexpression of lysyl-tRNA synthetase increases the concentration of tRNA^Lys^ in HIV-1 particles [117]. Although packaging tRNA^Lys^ by the respective synthetase would appear to be beneficial from an evolutionary point of view, since it ensures selective recruitment of reverse transcriptase primers, there are deeper issues to be considered. First, aminoacyl-tRNA synthetases in general have a high affinity towards their substrate. Thus, LysRS binds tRNA^Lys^ very tightly and so a release mechanism is required. Second, since the absolute number of primer tRNAs in virus particles is not high and there are only two copies of vRNA, the likelihood of primer binding to the specific primer binding site is rather low [118]. However, these potential problems are addressed by the existence of the tRNA anti-codon-like element (TLE) in the 5′-UTR of the viral RNA. This highly conserved motif is located upstream of the PBS and forms a stem-loop which has been shown by small-angle X-ray scattering (SAXS) to adopt an anticodon stem-loop-like tertiary structure [119]. Like the priming tRNA^Lys3^, the TLE has the “anticodon” UUU and can bind LysRS very efficiently. Moreover, the whole 5′-UTR, including TLE and two additional stem-loops downstream of the PBS, binds LysRS with nanomolar affinity, which is even higher than the affinity of LysRS for tRNA^Lys3^ [118] According to this proposed mechanism, tRNA^Lys^ gets displaced upon binding of TLE to the LysRS. Since this happens in close proximity to PBS, the released tRNA can efficiently bind with its 3′-terminus to the complementary primer binding site [118].

As mentioned above, the retrovirus MuLV uses tRNA^Pro^ for priming reverse transcription. However, this virus does not recruit prolyl-tRNA synthetase (ProRS) into the virus particles [115]. Still, a two- to four-fold enrichment of tRNA^Pro^ has been observed in MuLV particles [120]. This enrichment is achieved by translation of the viral RNA in proximity to the budding virus particle. The MuLV RNA contains a region at the frameshift site which is enriched in Pro, Arg and Gly codons. High usage of particular tRNAs to decode an mRNA region enriched for the respective codon increases the local concentration of these tRNAs while synthesizing the packaging proteins. In the case of MuLV, a co-translational enrichment of tRNA^Pro^, tRNA^Arg^ and tRNA^Gly^ is expected. Mutating the primer binding site of MuLV to accept tRNA^Arg^ or tRNA^Gly^ yields wildtype-like reverse transcription rates suggesting the enrichment of these tRNAs [121]. Interestingly, similar results have been obtained for HIV-1, by exchanging codons for tRNA^Lys3^ with those for the likes of tRNA^Lys1,2^. Mutants of this sort can use tRNA^Lys1,2^ efficiently for priming reverse transcription, allowing wildtype-like replication and infectivity [121]. It is noteworthy that since the synthetase recognizes all tRNA^Lys^ species, the composition in HIV particles reflects the relative tRNA^Lys^ concentrations of the host cell. However, only tRNA^Lys3^ is used to prime the reverse transcriptase reaction [116]. Interestingly, by using tRNA microarrays, enrichment of non-lysyl tRNAs has also been found in HIV-1 particles, namely tRNA^Asn^ and the rare tRNA^Ile^_UAU_ [122]. The latter tRNA is especially interesting, since it decodes AUA, which is the rarest isoleucine codon in human cells (17%), but the most frequent isoleucine codon in the HIV-1 genome (54%) [123]. In line with this, tRNAs in virus particles correlate well with the codon usage in late genes (gag, pol, env, vif, vpu and vpr), but not with early genes (tat, rev and nef) [124,125], assuming a co-translational packaging of highly used tRNAs in the translation of viral proteins. Similar results have been obtained from vaccinia and influenza A virus: six hours post-infection tRNAs have been purified from polysomes, including ribosome-bound and tRNAs associated with aminoacyl-tRNA synthetases. Those polysomal tRNAs have mirrored the codon usage of the viruses. Strikingly, the correlation has been more significant for vaccinia than for influenza A, consistent with a more complete shutdown of host proteins and a higher expression of viral proteins in vaccinia virus [11]. These results are of general interest for studies of locally restricted translation of certain mRNAs, e.g., in synapses—suggesting a local tRNA pool, which matches the codon usage of tRNAs being used immediately in translation.

## 5. tRNA Modifications

One of the striking features of tRNAs are their post-transcriptional modifications, which are listed in the RNA Modification Database [126,127,128]. Modifications range from a rather simple methylation to modifications with bulky groups that include amino acid (e.g., lysidine, k2C) or sugar (e.g., galactosyl-queuosine, galQ) structures. There is a broad variety of different functions that can be summarized in three general rules. First, modifications in or around the anticodon loop affect translation. Second, modifications in the body affect tRNA folding and stability. Third, modifications at various positions ensure tRNA recognition by aminoacyl transferases [129]. Recent findings furthermore have shown that modifications additionally regulate susceptibility of tRNAs to ribonuclease degradation [130] and thus might regulate the biogenesis of different tRNA fragments with regulatory functions [131]. While concentrations of tRNAs are rather constant in a given cell due to costly biosynthesis and long half life, modifications can change rather quickly upon changes of environmental conditions, e.g., upon oxidative stress, approximately one third of yeast tRNA modifications have been found altered, whereas variations have ranged from five-fold reduction to four-fold increase in modifications and have revealed specific patterns depending on the toxic agent applied [132]. By a combination of HPLC and mass spectrometry, an array of 23 modifications can be quantitatively measured [132].

As mentioned above, modifications in the tRNA anticodon can regulate decoding during protein biosynthesis. Thiolation of the uridine 34 (U34) in the anticodon of tRNA^Lys^_UUU_, tRNA^Glu^_UUC_ and tRNA^Gln^_UUG_ allows those tRNAs to wobble. For example, as a result of the thio-modifications s^2^U or mcm^5^s^2^U tRNA^Lys^_UUU_ is able to decode both lysine codons AAA and AAG [133,134,135]. Interestingly, in yeast, thiolation of those tRNAs depends on sulfur amino acid supply [136] and is reduced at elevated temperatures [137]. In *E. coli*, alterations of thiolation of tRNA^Lys^_UUU_ lead to altered susceptibility against lambda phage infection. Hypomodification leads to decreased infection of the virus [138]. Lambda phage replication depends on a particular ratio between the proteins gpG and the (–1)-frameshift product gpGT. The frameshift event occurs at the slippery site GGGAAAG, which, in both frames, encodes glycine and lysine. Hypomodification of tRNA^Lys^_UUU_ causes increased frameshifting and thus changes the gpG:gpGT ratio, resulting in lower infectivity of the lambda phage [138]. It remains elusive whether hypomodified tRNA^Lys^ directly facilitates frameshifting, which is counterintuitive, since the modification broadens codon specificity, or whether hypomodification leads to lower charging levels [139,140]. This could stall the ribosome at the slippery site and thus enhance the chance for (–1) frameshifting as has been shown in the gene encoding huntingtin [141]. Modifications in the anticodon loop of the human tRNA^Lys3^ have been shown to be important for HIV-1. The tRNA^Lys3^ bears 5-methoxycarbonylmethyl-2-thiouridine (mcm^5^s^2^U_34_) at the wobble position of the anticodon and 2-methylthio-N6-threonylcarbamoyladenosine (ms^2^t^6^A_37_) at the nucleotide 3′-adjacent to the anticodon, a combination that is unique among all tRNAs in human cells [142,143]. As described above, Ncp7 molecules bind the tRNA and the 5′-UTR of the viral RNA to melt stem loop structures in order to allow annealing and subsequent reverse transcription. The modified anticodon stem loop (ASL^Lys^) has shown ten-times higher affinity for Ncp7 than the unmodified ASL, identifying post-transcriptional modifications as important discrimination factors for Ncp7 binding and consequently, tRNA annealing, packaging and priming reverse transcription [144,145]. Using evolutionary algorithms the same group has identified and subsequently tested peptides that specifically recognize modified tRNA^Lys3^ [145,146]. Those peptides cannot only be used to study protein:RNA interactions but also as therapeutics against HIV-1 [145].

## 6. Conclusions and Perspectives

Transfer RNAs play various roles in the life cycle of viruses. They not only serve the canonical function of delivering amino acids to the ribosome for protein synthesis but serve virus-specific functions in regulating translation, packaging and priming reverse transcription. In many cases, not only concentration but also aminoacylation and modification of particular tRNA species play a crucial role in those functions. On the one hand, viruses use host tRNAs for their purposes, in some cases specifically packaging them into virions. On the other hand, many viruses encode their own tRNAs to be more independent of their host. Recent advances in the global analysis of tRNA concentrations, now reaching nearly single nucleotide resolution, allow analysis of tRNA pools from virus particles and host cells at different temporal and spatial conditions. Additionally, evolution of viruses has created fascinating mechanisms and pathways which broaden the functional range of tRNAs beyond their role as simple amino acid:codon adaptors.
